# The biomechanical effect on the adjacent L4/L5 segment of S1 superior facet arthroplasty: a finite element analysis for the male spine

**DOI:** 10.1186/s13018-021-02540-0

**Published:** 2021-06-17

**Authors:** Zewen Shi, Lin Shi, Xianjun Chen, Jiangtao Liu, Haihao Wu, Chenghao Wang, Zeming Chen, Fang Yang, Sheng Yu, Qingjiang Pang

**Affiliations:** 1 Ningbo University School of Medicine, Ningbo, China; 2 Department of Orthopaedics, Hwa Mei Hospital, The Affiliated Hospital of University of Chinese Academy of Science, Ningbo, 315010 China

**Keywords:** Superior facet, Finite element, Range of motion, Stress

## Abstract

**Background:**

The superior facet arthroplasty is important for intervertebral foramen microscopy. To our knowledge, there is no study about the postoperative biomechanics of adjacent L4/L5 segments after different methods of S1 superior facet arthroplasty. To evaluate the effect of S1 superior facet arthroplasty on lumbar range of motion and disc stress of adjacent segment (L4/L5) under the intervertebral foraminoplasty.

**Methods:**

Eight finite element models (FEMs) of lumbosacral vertebrae (L4/S) had been established and validated. The S1 superior facet arthroplasty was simulated with different methods. Then, the models were imported into Nastran software after optimization; 500 N preload was imposed on the L4 superior endplate, and 10 N⋅m was given to simulate flexion, extension, lateral flexion and rotation. The range of motion (ROM) and intervertebral disc stress of the L4-L5 spine were recorded.

**Results:**

The ROM and disc stress of L4/L5 increased with the increasing of the proportions of S1 superior facet arthroplasty. Compared with the normal model, the ROM of L4/L5 significantly increased in most directions of motion when S1 superior facet formed greater than 3/5 from the ventral to the dorsal or 2/5 from the apex to the base. The disc stress of L4/L5 significantly increased in most directions of motion when S1 superior facet formed greater than 3/5 from the ventral to the dorsal or 1/5 from the apex to the base.

**Conclusion:**

In this study, the ROM and disc stress of L4/L5 were affected by the unilateral S1 superior facet arthroplasty. It is suggested that the forming range from the ventral to the dorsal should be less than 3/5 of the S1 upper facet joint. It is not recommended to form from apex to base.

**Level of evidence:**

Level IV

## Introduction

In recent years, percutaneous transforaminal endoscopic discectomy (PTED) has been accepted as an alternative treatment for disc herniation due to its advantages over traditional open surgery [1–3]. However, the working channel is often difficult to establish in L5/S1 segment as high iliac crest, hyperplastic articular process and narrow foramen intervertebrale. In these cases, the articular process arthroplasty is needed [4]. Facetectomy is an effective procedure of PTED for the enlargement of operational space and for the decompression of stenosis nerve roots. In the long term, the degeneration of responsible segment and adjacent segments are clinically common after facet arthroplasty. Though the effect of S1 superior articular process arthroplasty on responsible segment had been reported [5], the effect on adjacent segment (L4/L5) has not been unreported.

Medical finite element method (FEM) is a technique of reconstructing three-dimensional model from image data. Through the digital simulation of various operations, the stress and displacement changes can be obtained. It has been widely used in the research of the bone, joint and other fields. In this study, FEM was used to simulate S1 superior articular process arthroplasty. The S1 superior articular process was formed parallel to the S1 upper endplate from the apex to the base and perpendicular to the S1 upper endplate from the ventral to the dorsal. The effect of adjacent segment (L4/L5) biomechanical change was explored.

## Materials and methods

### Research object and data collection

Eight healthy male volunteers were selected, whose age range from 22 to 29 years old. X-ray of the lumbosacral vertebra was taken to exclude pathological conditions. Computed tomography scan of the lumbar spine was obtained with 1.0 mm thickness. The work has been approved by the Hospital Ethical Committee (Hwa Mei Hospital, University of Chinese Academy of Science) and that subjects gave informed consent to the work.

### Normal finite element model

The computed tomography images were post-processed for boundary detection with the Mimics 17.0 (Materialise, Belgium), and then geometric models were established. Geomagic Studio 10.0 (Geomagic, USA) was used to import the spine modes for repair and noise reduction. Hypermesh 13.0 (Altair, USA) was used to generate the FE mesh for analysis. Nastran 2012 (MSC, USA) was used to construct finite element models (FEMs). The material properties of the model were listed in Table 1 using the results of previously published studies [1, 6, 7].

**Table 1:** Material properties of FE models

Component	Element type	Elastic modulus (MPa)	Poisson ratio
Cortical bone	lsotropic, elastic tetra element	12000.0	0.30
Cancellous bone	lsotropic, elastic tetra element	100.0	0.20
End plate	Nonlinear spring element	2000.0	0.20
Fibres of annulus fibrosis	Rebar	92.0	0.45
Matrix of annulus fibrosis	Neo-Hookean, hex element	4.2	0.45
Nucleus pulposus	Incompressible fluid element	1.0	0.50
Anterior longitudinal ligament	Tension only, truss element	7.8	0.30
Posterior longitudinal ligament	Tension only, truss elements	10.0	0.30
Supraspinous ligament	Tension only, truss elements	8.0	0.30
Interspinous ligament	Tension only, truss elements	8.0	0.30
Ligamentum flavum	Tension only, truss elements	10.0	0.30
Intertransverse ligament	Tension only, truss elements	10.0	0.30
Capsular ligament	Tension only, truss elements	15.0	0.30
Articular cartilage	Nonlinear spring element	25.0	0.40
Articuli intervertebrales	Sliding surface to surface contact	10.0	0.30

### The establishment of the facet arthroplasty finite element model

**Fig. 1:**
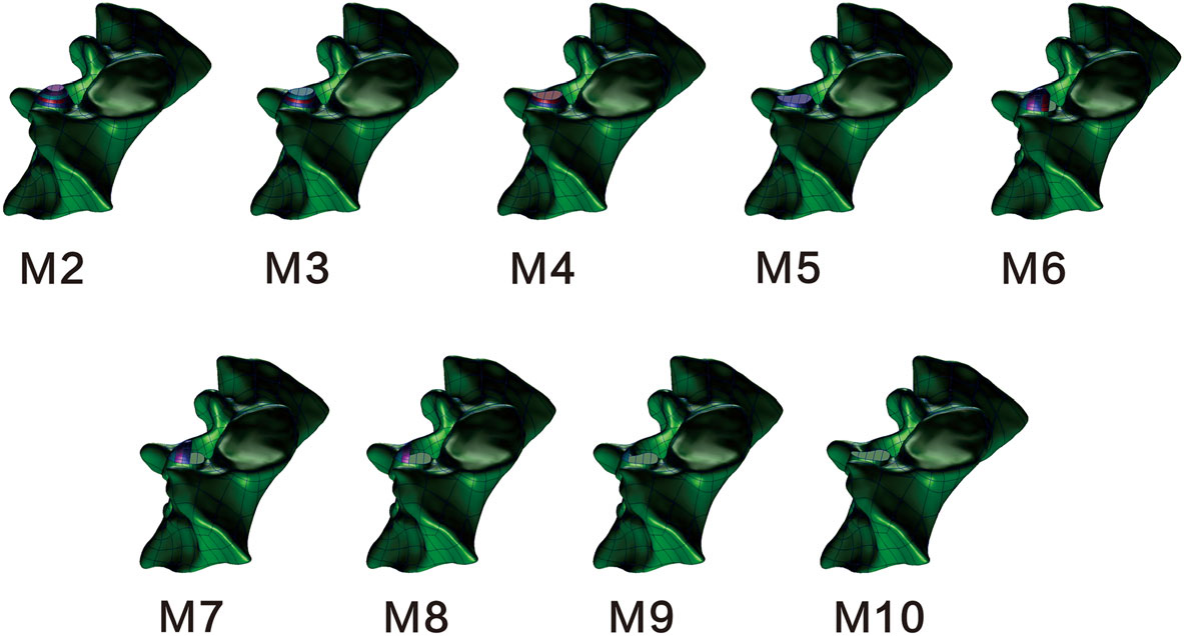
Schematic diagram of S1 superior facet arthroplasty

The surgical models were constructed based on the validated intact model (M1). Geomagic was used to simulate unilateral S1 superior articular process arthroplasty. The S1 superior articular process was graded formed (1/5, 2/5, 3/5, 4/5, 5/5) parallel to the S1 upper endplate from the apex to the base (transverse plasty) and perpendicularly to the S1 upper endplate from the ventral to the dorsal (longitudinal plasty), respectively. The models established were defined as M2-M10 (M2-M5 represents transverse plasty 1/5-4/5, M6-M9 represents longitudinal plasty 1/5-4/5, and M10 represents plasty 5/5) (Fig. 1).

### Boundary and loading conditions

**Fig. 2:**
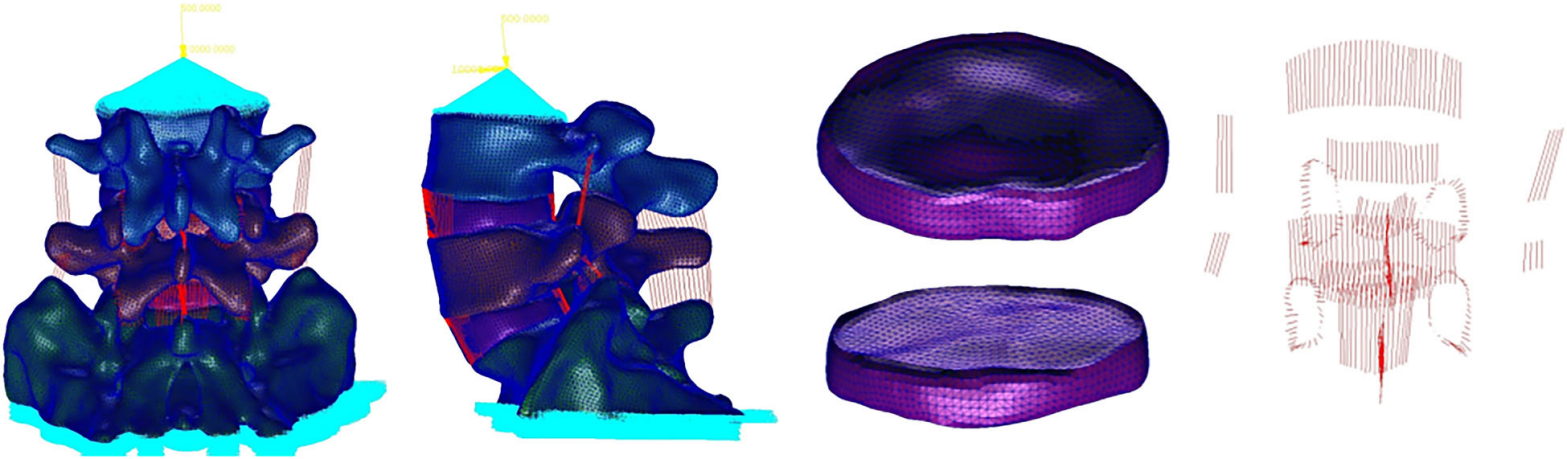
Normal lumbosacral vertebral finite element model

The inferior surface of the S1 vertebra was constrained completely (Fig. 2). A vertical load of 500 N and a torque of 10 N⋅m were applied to the L4 to simulate the weight of the body and various loading conditions of the lumbar spine. The torque along the axis generate flexion, extension, forming contralateral flexion, forming side flexion, forming contralateral rotation and forming side rotation. The ROM and intervertebral disc von Mises stress of adjacent segment (L4/L5) were quantified.

### Statistical analysis

The SPSS 19.0 software was adopted for statistical analysis in this study. Data was represented by x ± s. ANOVA was used between groups, and Dunnett’s analysis was used for pairwise comparison. Herein, P < 0.05 was considered to be statistically significant.

## Results

### Validation of the FEM

**Fig. 3:**
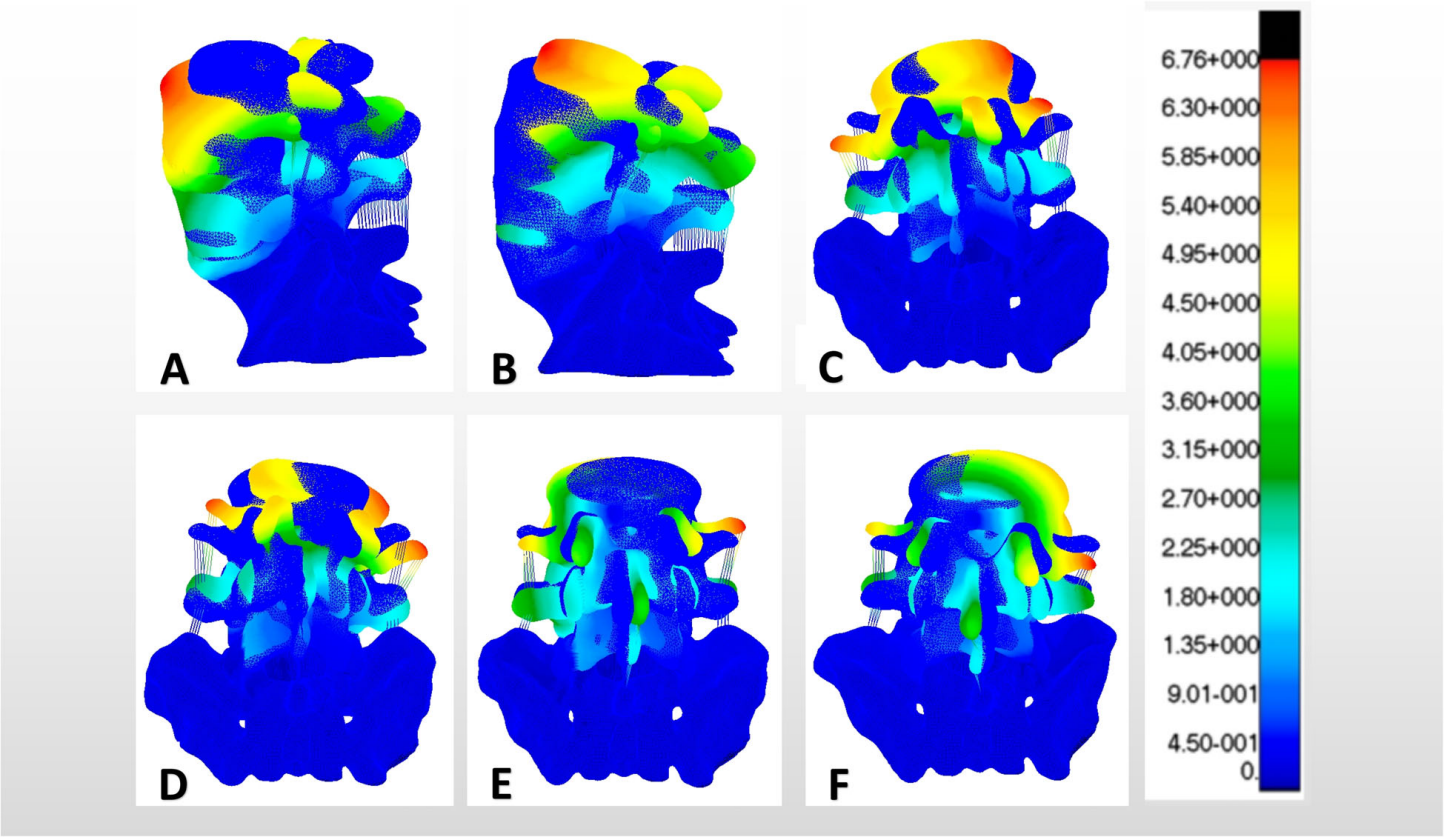
Normal model displacement nephogram

The displacement nephogram of normal model was acquired (Fig. 3). Intact FEMs were validated by comparing the ROM of the L4/L5 and L5/S1 with the results of the test performed by Yamamoto and Zhitao Xiao [6, 7]. The ROM of the intact FEMs was in the range of reported data [6, 7], and the result had been published in previous study by our research group [5] (Table 2).

**Table 2:** The dates of intervertebral ROM were compared with previous studies

	This study			Yamamoto’s study			Zhitao Xiao’s study		
	Flexion extension	Lateral flexion	Lateral rotation	Flexion extension	Lateral flexion	Lateral rotation	Flexion extension	Lateral flexion	Lateral rotation
**L4-L5**	12.19 ± 2.61	11.46 ± 1.53	5.09 ± 1.22	14.8 ± 2.10	12.2 ± 2.25	3.7 ± 1.50	14.20	13.23	4.23
**L5-S1**	14.67 ± 3.37	11.32 ± 1.85	3.06 ± 1.70	16.9 ± 2.05	11.3 ± 2.35	2.5 ± 0.75	17.29	12.56	2.70

### ROM of L4/L5 segment

**Fig. 4:**
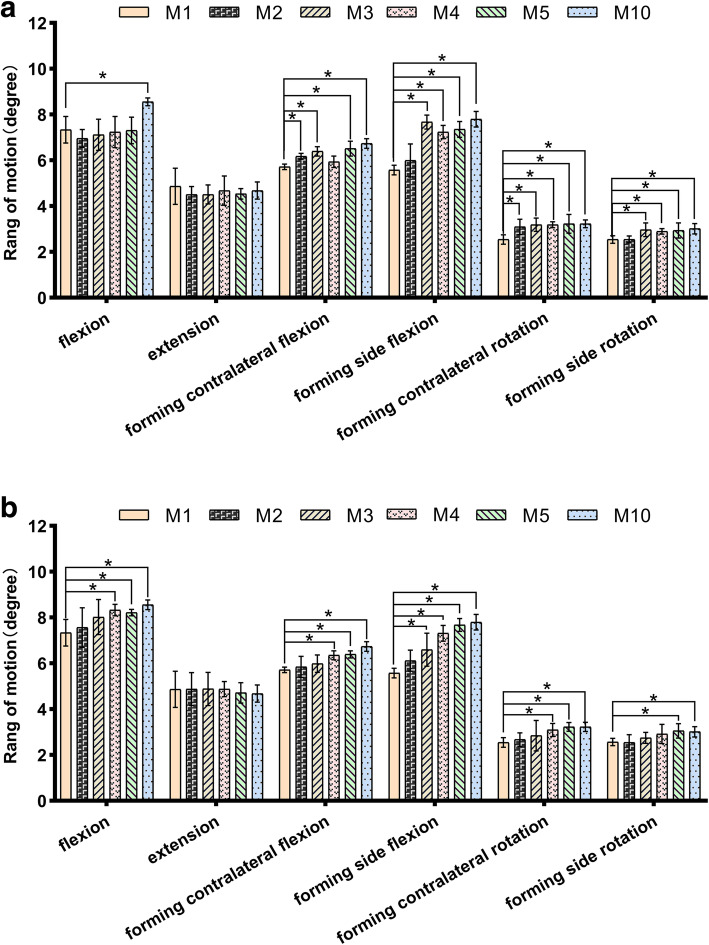
**a** Lumbar motion of L4/L5 segment after S1 superior facet transverse arthroplasty; *p<0.05. **b** Lumbar motion of L4/L5 segment after S1 superior facet longitudinal arthroplasty; *p<0.05

In transverse forming, ROM of L4/L5 increased significantly in the lateral flexion and lateral rotation. The growth rates of forming contralateral flexion, forming side flexion, forming contralateral rotation and forming side rotation were 8–18%, 4–35%, 22–27% and 0–18%, respectively (Fig. 4a). In longitudinal forming, ROM of L4/L5 increased significantly in flexion, lateral flexion and axial rotation. The growth rates of flexion, forming contralateral flexion, forming side flexion, forming contralateral rotation and forming side rotation were 3–17%, 2–18%, 6–35%, 5–27% and 0–18%, respectively (Fig. 4b).

### Intervertebral disc stress of L4/L5 segment

**Fig. 5:**
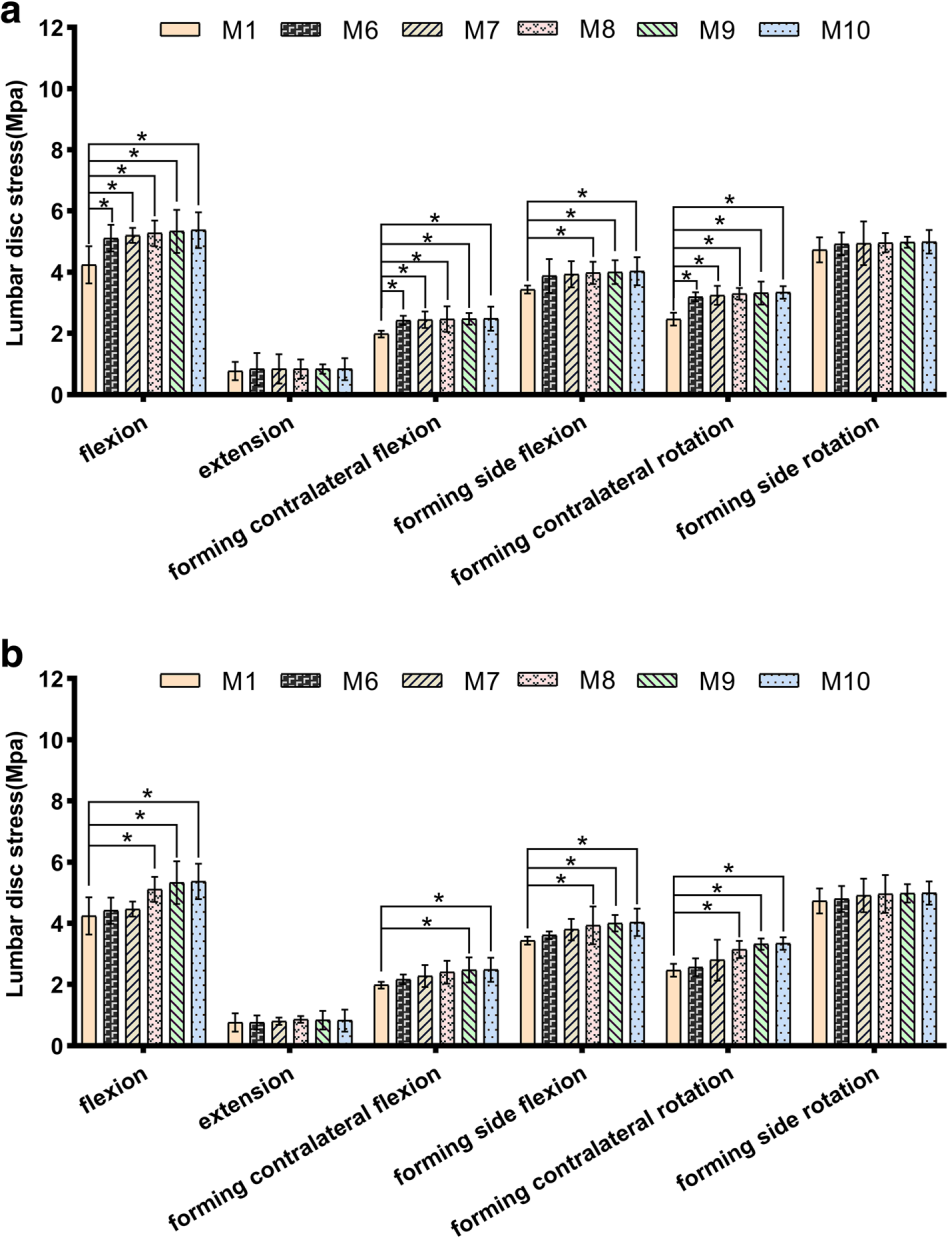
**a** Lumbar disc stress of L4/L5 segment after S1 superior facet transverse arthroplasty; *p<0.05. **b** Lumbar disc stress of L4/L5 segment after S1 superior facet longitudinal arthroplasty; *p<0.05

In longitudinal and transverse forming, intervertebral disc stress of L4/L5 increased significantly in flexion, lateral flexion and forming contralateral rotation. In transverse forming, the growth rates of flexion, forming contralateral flexion, forming side flexion and forming contralateral rotation were 20–27%, 22–25%, 13–17% and 30–36%, respectively (Fig. 5a). In longitudinal forming, the growth rates of flexion, forming contralateral flexion, forming side flexion and forming contralateral rotation were 4–27%, 9–25%, 5–17% and 4–36%, respectively (Fig. 5b).

## Discussion

Due to the anatomical characteristics of L5/S1 segment, posterior approach is generally the preferred method of spinal endoscopy. The results show that posterior arthroplasty can maintain clinical improvement and radiological stability [8]. However, the posterior approach is difficult for patients with stenosis of vertebral canal, small inter-laminar space and extreme lateral lumbar disc herniation. In addition, research has shown that the lumbar instability would occur after the medial of inferior articular process is removed more than 1/2 through the posterior approach [9]. At this time, the posterolateral approach is needed. However, in L5/S1 segment, the superior articular process arthroplasty is usually needed to enlarge the foramen intervertebrale under posterolateral approach so as to reduce the interference in nerves and expand the surgical indications [10]. Unilateral S1 superior articular process reconstruction has a great impact on the biomechanics of the responsible segment, and the results have been published in previous studies by our research group [5]. Adjacent disc degeneration often occurs after PTED, and the annual risk rate of clinically related adjacent segmental diseases is reported to be 0.6–3.9% [11, 12]. The main reason is the biomechanical effect of facetoplasty on adjacent segments [13]. In general, the partial resection of the superior articular process via the superior articular process (SAP) approach is safe [14]. However, there has been no consensus about the effects of S1 superior articular process forming on the biomechanics of adjacent L4/L5 segment. Hence, it is of great significance to explore the effect of S1 superior facet arthroplasty on the biomechanics of L4/L5 through finite element analysis and indirectly reflect the effect of S1 superior articular process arthroplasty on the risk of adjacent segment degeneration.

**Fig. 6:**
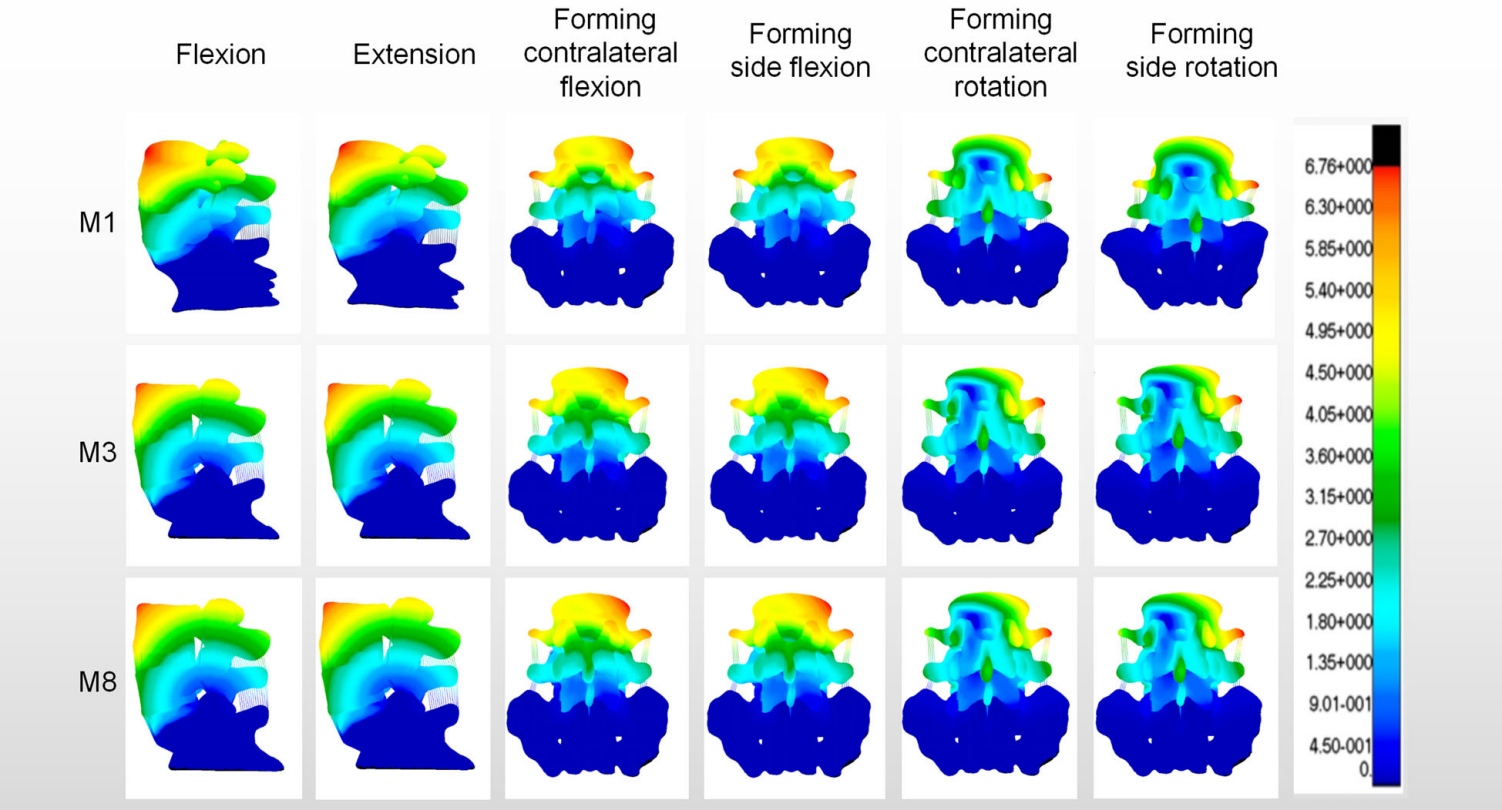
The strain nephogram of biomechanical characteristics in M1, M3 and M8

The biomechanical study of facet joint and spinal degeneration has been deepened gradually, with the popularity of the technology of PTED. Matsuo et al. [15] showed that the degenerative lumbar spondylolisthesis is significantly related to the sagittal and axial angles of the facet joints. It can be seen that the facet joint plays an important role in spinal degeneration. As we know, facet arthroplasty can reduce the stability and increase the risk of degeneration of the responsible segment [5, 16, 17]. However, the effect of S1 superior articular process arthroplasty on the ROM of adjacent L4/L5 segment has not been reported. The results of this study showed (Fig. 6) that the ROM of L4/L5 segment increased significantly in flexion, lateral flexion and lateral rotation, when the longitudinal forming was more than or equal to 3/5. Compared with the normal model, the difference was statistically significant. In lateral bending and lateral rotation, the ROM of L4/L5 segment increased significantly, when the transverse forming was more than or equal to 2/5. Compared with the normal model, the difference was statistically significant. These results suggest that the ROM of adjacent L4/L5 segment would be affected after the longitudinal shape of S1 superior articular process is more than or equal to 3/5 or the transverse shape is more than or equal to 2/5. The reason for this effect is that the anterior aspect of the L5 body has a greater height compared to the posterior. The reason for this obvious effect is the Spine Sacral Angle (SSA) between the lumbar spine and sacrum, and L5/S1 plays an important role in the SSA due to its anatomical characteristics. This determines that the mechanical changes of L5/S1 joint have a significant impact on the adjacent segments of the lumbar spine [18].

**Fig. 7:**
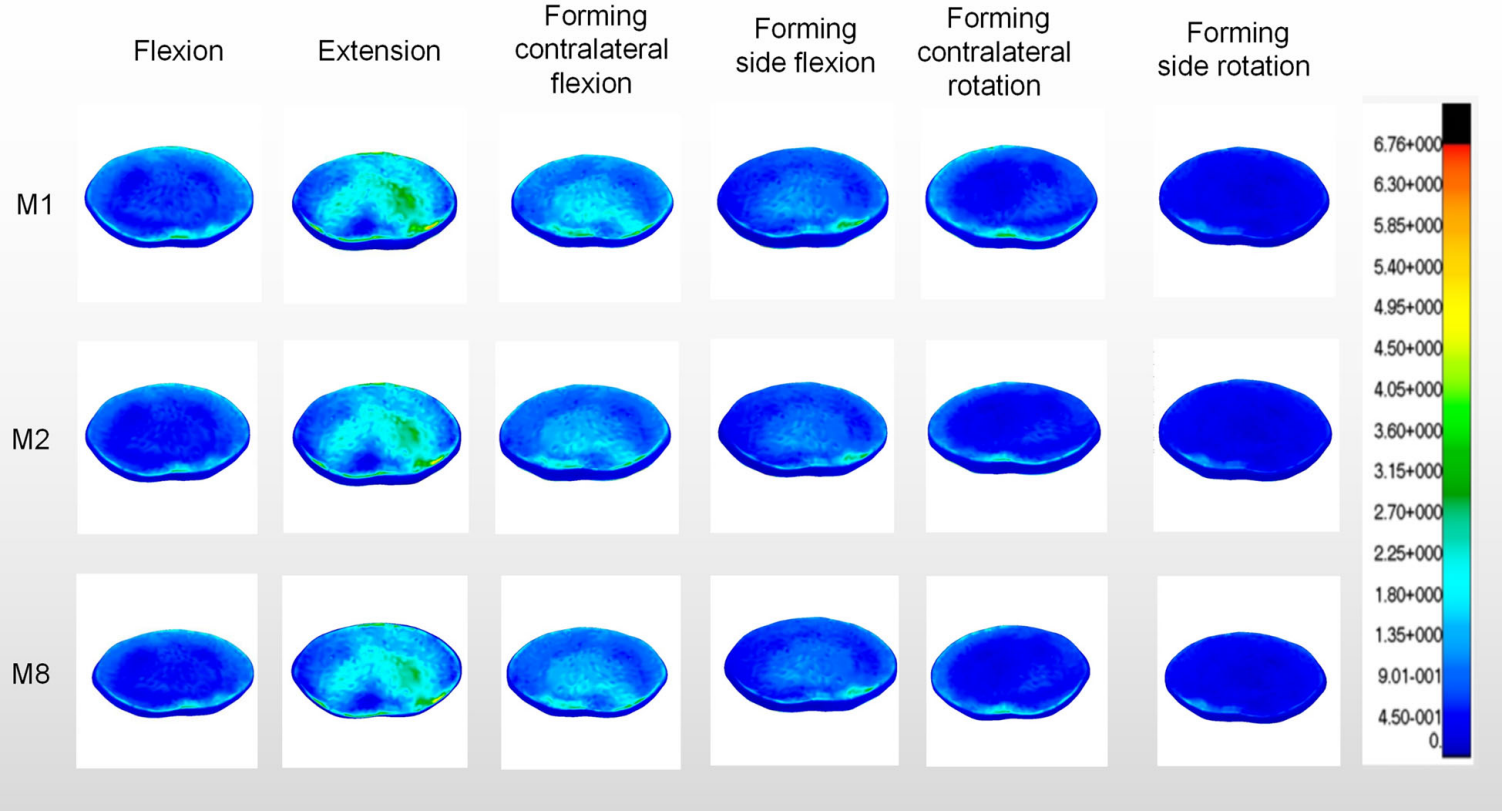
The stress nephogram of biomechanical characteristics in M1, M2 and M8

As we know, 25% of the axial compressive stress and 40–65% of the rotational and shear stress of the lumbar spine are borne by the facet joint [19]. The increased stress of the intervertebral disc caused by the asymmetry of the articular process contributes to the increased risk of lumbar degeneration [20]. Qian et al. [17] reported that 1/4 of L5 unilateral superior articular process forming could increase the stress of the same segment of intervertebral disc. In terms of adjacent segments, studies have shown that in L4/L5 segments, quarter facet arthroplasty has no significant effect on adjacent segments. However, significant stress changes occurred in the half facet resection model [21]. However, few studies about the effect of S1 superior facet arthroplasty on the adjacent disc stress have been reported. The results of this study showed (Fig. 7) that the disc stress of L4/L5 segment increased significantly in flexion, contralateral flexion and contralateral rotation after the longitudinal forming was more than or equal to 3/5. Compared with the normal model, the difference was statistically significant. In flexion, extension, lateral flexion and contralateral rotation, the disc stress of L4/L5 segment increased significantly after the transverse forming was more than or equal to 1/5. Compared with the normal model, the difference was statistically significant. It was worth noting that the stress of the L4/L5 disc increased most obviously when it rotated to the contralateral side of the forming. This is consistent with the statement that the facet joint plays a major role in the torsional stiffness of the intervertebral disc [22]. In other words, the disc stress of adjacent L4/L5 segment would be affected after the longitudinal form of S1 upper joint is more than or equal to 3/5 or the transverse form is more than or equal to 1/5, resulting in an increased risk of degeneration of adjacent segment.

In terms of experimental methods, three-dimensional finite element method has been widely used in orthopaedic field, especially as a high simulation method in spine [23]. However, in another way, the accuracy of the research is decreased by the finite element method owing to it is a method of simplifying the complexity. In addition, this study infers the relationship between facet arthroplasty and adjacent segment degeneration from the immediate influence, failing to monitor the whole degeneration process dynamically. In the experimental design, the apex and basal part are common locations for S1 superior articular process arthroplasty. In addition, the original shape of foramen intervertebrale should be maintained and the destruction of anatomical structure should be reduced as far as possible. Two experimental methods were designed, parallel to S1 upper endplate from the top to the base and perpendicular to S1 upper endplate from ventral to dorsal under the premise of taking the upper edge of S1 pedicle as the lowest level.

## Conclusions

In conclusion, the S1 superior articular process of the lumbar spine is not only of great significance to the biomechanics of the same segment, but also to the adjacent segment. It is possible that the stability of adjacent L4/L5 segment would be decreased and the stress of intervertebral disc would be increased after unilateral S1 superior facet arthroplasty. It is suggested to form the ventral to the dorsal of unilateral S1 superior articular process arthroplasty which should be controlled less than or equal to 3/5, and it is not recommended to form from the apex to the base, combining with the previous study of the effect of S1 superior articular process arthroplasty on the same segment [5]. Otherwise, the long-term risk of adjacent segment degeneration would be increased.

## Data Availability

All data and material are fully available.

## Abbreviations

FEM: Finite element method
FEMs: Finite element models
ROM: Range of motion
PTED: Percutaneous transforaminal endoscopic discectomy
